# Parental perceptions of children's oral health: The Early Childhood Oral Health Impact Scale (ECOHIS)

**DOI:** 10.1186/1477-7525-5-6

**Published:** 2007-01-30

**Authors:** Bhavna Talekar Pahel, R Gary Rozier, Gary D Slade

**Affiliations:** 1 Department of Health Policy and Administration, School of Public Health, University of North Carolina at Chapel Hill, Chapel Hill, NC, USA; 2 Australian Research Centre for Population Oral Health, Dental School, 1st floor, 122 Frome St, University of Adelaide, SA 5005, Australia

## Abstract

**Background:**

Dental disease and treatment experience can negatively affect the oral health related quality of life (OHRQL) of preschool aged children and their caregivers. Currently no valid and reliable instrument is available to measure these negative influences in very young children. The objective of this research was to develop the Early Childhood Oral Health Impact Scale (ECOHIS) to measure the OHRQL of preschool children and their families.

**Methods:**

Twenty-two health professionals evaluated a pool of 45 items that assess the impact of oral health problems on 6-14-year-old children and their families. The health professionals identified 36 items as relevant to preschool children. Thirty parents rated the importance of these 36 items to preschool children; 13 (9 child and 4 family) items were considered important. The 13-item ECOHIS was administered to 295 parents of 5-year-old children to assess construct validity and internal consistency reliability (using Cronbach's alpha). Test-retest reliability was evaluated among another sample of parents (N = 46) using the intraclass correlation coefficient (ICC).

**Results:**

ECOHIS scores on the child and parent sections indicating worse quality of life were significantly associated with fair or poor parental ratings of their child's general and oral health, and the presence of dental disease in the child. Cronbach's alphas for the child and family sections were 0.91 and 0.95 respectively, and the ICC for test-retest reliability was 0.84.

**Conclusion:**

The ECOHIS performed well in assessing OHRQL among children and their families. Studies in other populations are needed to further establish the instrument's technical properties.

## Background

Numerous measures have been developed in recent years to assess the effect of oral health problems on individuals' physical, mental and social health and well-being [1]. These instruments place emphasis on assessing people's subjective experiences with health and disease states, both treated and untreated. They reflect a move within dentistry toward a more holistic model of health, rather than a mechanistic view that sees the individual as existing independent of his or her environment. Further, this concept of oral health-related quality of life (OHRQL) falls within the domains of an "outcomes" model, which emphasizes consideration of people's self-reports in addition to the traditional disease and diagnosis oriented "biomedical" model [2].

Instrument development in the area of OHRQL initially focused on adult and geriatric populations [3]. More recently, interest has centered on assessing the OHRQL of children and adolescents. Canadian researchers have developed the Child Oral Health Quality of Life (COHQoL) questionnaires, which include the Parental-Caregiver Perceptions Questionnaires (P-CPQ) and the Family Impact Scale (FIS) for children aged 6–14 years, and three Child Perceptions Questionnaires for children aged 6 to 7 (CPQ_6–7_), 8 to 10 (CPQ_8–10_), and 11 to 14 (CPQ_11–14_) years of age [4-7]. All questionnaires, except the CPQ_6–7 _have been developed and tested. Few instruments exist for the assessment of the impact of oral disease on the quality of life of children. Filstrup and colleagues [8] used the Michigan OHRQL Scale to examine the effect of treating early childhood caries (ECC) on children's quality of life. The children in this study ranged in age from 22–70 months and the authors obtained both child self-reports (limited to children 36–70 months of age) and parent reports of their child's OHRQL. No instruments are available to measure   the impact of ECC on children or their families, and the Michigan OHRQL scale has undergone only limited testing in a clinical setting. Further, no instrument designed specifically for use in epidemiological surveys is currently available to assess the OHRQL of children of preschool age.

About 1 in 5 preschool children in the United States experience dental disease in the form of early childhood caries (ECC) [9]. Compared to those children who do not have dental caries early in life, those who do are more likely to have repeat episodes as they become older children and adolescents [10]. ECC causes pain in a significant number of children, and also can interfere with the growth of the body, with adverse effects on body weight and height and can result in failure to thrive [11-13]. Responsibility for the health of young children is usually borne by adults. Also, adults generally make decisions about their children's health. Therefore, assessing parents' perceptions about how oral health problems, including symptoms, disease and its treatment influence their children's quality of life is important. Evidence also indicates that ECC results in lost workdays for caregivers who have to stay at home to take care of their child, or spend time and money in accessing dental care [14]. Thus, these influences on caregivers also are important to measure as part of assessing young children's OHRQL.

Evidence from the child development and psychology literature indicates that children younger than 6 years of age are unable to accurately recall everyday and unique events beyond 24 hours. Children begin to reason about the timing of past events with respect to the day of the week, month or season at the age of 7 or older [15]. In addition, only at about 6 years of age do children become capable of abstract thinking, which likely underlies many perceptions of health and disease [16]. Research that has attempted to use preschool age children as respondents in OHRQL studies has met with limited success [8]. All of these developmental characteristics of children mean that adults must report impacts of dental disease in these children.

In this paper we describe the development of the Early Childhood Oral Health Impact Scale (ECOHIS) (Table 1) to assess the impact of oral health problems and related treatment experiences on the quality of life of preschool age children (3 to 5 years old) and their families. The objective was to develop a short instrument to be completed by the child's parent or primary caregiver for use in epidemiological surveys to discriminate between children with and without dental disease experience.

**Table 1:** The Early Childhood Oral Health Impact Scale (ECOHIS)

"*Problems with the teeth, mouth or jaws and their treatment can affect the well-being and everyday lives of children and their families. For each of the following questions please circle the number next to the response that best describes your child's experiences or your own. Consider the child's entire life from birth until now when answering each question. If a question does not apply, check 'Never*"' *Response options: 1. Never, 2. Hardly ever, 3. Occasionally, 4. Often, 5. Very often and 6. Don't know*.
1. How often has your child had **pain **in the teeth, mouth or jaws? *(Child symptoms domain)*
How often has your child......because of dental problems or dental treatments? *(Child function domain)*
2. had **difficulty drinking hot or cold beverages**
3. had **difficulty eating some foods**
4. had **difficulty pronouncing any words**
5. **missed preschool, daycare or school**
How often has your child......because of dental problems or dental treatments? *(Child psychological domain)*
6. had **trouble sleeping**
7. been **irritable or frustrated**
How often as your child......because of dental problems or dental treatments? *(Child self-image/social interaction domain)*
8. **avoided smiling or laughing **when around other children
9. **avoided talking **with other children
How often have you or another family member......because of your child's dental problems or dental treatments? *(Parent distress domain)*
10. been **upset**
11. felt **guilty**
How often.... *(Family function domain)*
12. have you or another family member **taken time off from work **.....because of your child's dental problems or dental treatments
13. has your child had dental problems or dental treatments that had a **financial impact **on your family?

## Methods

### Overview of ECOHIS development and testing

**Figure 1:**
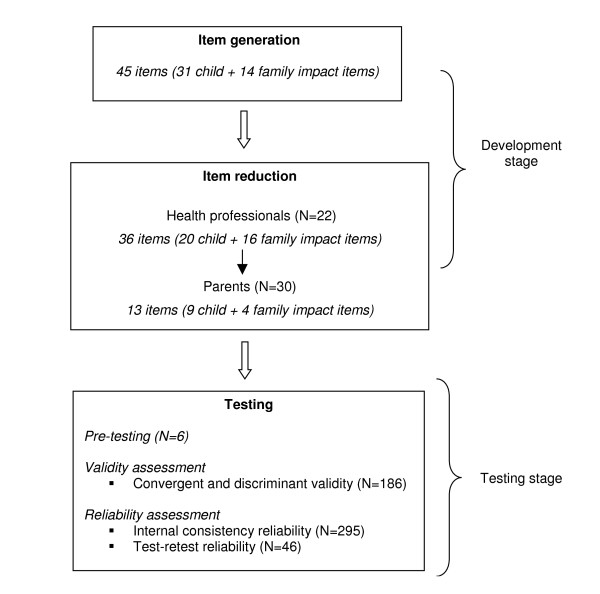
Steps in the development of the Early Childhood Oral Health Impact Scale (ECOHIS).

We used the methodology for developing and testing health-related quality of life instruments described by Juniper et al. [17] and Guyatt et al. [18] and procedures for scale development described by DeVellis [19] (see Figure 1). The *development *stage involved item generation (using the initial 45 item-pool provided by Jokovic and Locker, and a literature review) and item reduction (based on input from 22 health professionals and 30 parents/caregivers). The development stage was followed by the *testing *stage, which included pre-testing, and assessment of construct validity (convergent and discriminant) and reliability (internal consistency and test-retest) of the ECOHIS. The School of Public Health Institutional Review Board for Research Involving Human Subjects of the University of North Carolina at Chapel Hill approved all parts of this study.

### Item generation

We used a pool of 45 impact items provided to us by Drs. Jokovic and Locker for the initial item pool. This item pool was generated for development of the Parental-Caregiver Perceptions Questionnaires (P-CPQ) by Jokovic et al. [4] through focus groups, unstructured interviews and item-impact studies with parents of children 6 to 14 years of age. These 45 items (31 child and 14 family items) represented descriptive domains of symptom, function, emotional and family/social well-being. Many of the Jokovic et al. (2003) items are similar to those included in the Parent form of the Child Health Questionnaire (CHQ) [20] and the Infant Toddler Quality of Life Questionnaire (ITQOL) [21] developed by Landgraf and colleagues for children and adolescents 5-to-18 years of age and for infants and toddlers, respectively. We also reviewed generic and non-dental disease-specific quality of life instruments for preschool children to identify items relevant to children's oral health that were possibly missing from the 45-item pool. Because items identified from the literature review overlapped with those identified by Jokovic and colleagues (2003), only the items from the latter were used in the development of the ECOHIS.

### Item reduction

To assess relevance of the 45 items to preschool age children, we solicited opinions from a convenience sample of 22 health professionals who work with young children and their families on a routine basis or are researchers in dental public health. The respondents included three pediatric dentists in private practice, five pediatric dentists in academia, five front office/reception staff in private dental offices, three pediatricians, one developmental psychologist and five public health dentists. The respondents were asked to indicate, on a visual analog scale (VAS), their opinion of the degree of relevance ("Not at all relevant" to "Entirely relevant") of each item to children of preschool age. Based on responses and feedback from the health professionals, we developed a modified pool of 36 items by rewording, combining, or excluding irrelevant items. For example, the question that asked parents if their child had missed school because of dental problems was modified to ask whether the child had missed preschool, daycare or school.

The modified pool of 36 items was administered to a convenience sample of 30 parents of children 3 to 5 years of age with a range of dental care needs. Ten parents of children with low-to-moderate treatment needs were sampled from the graduate clinic of the Department of Pediatric Dentistry at the University of North Carolina at Chapel Hill (UNC) School of Dentistry. We also enrolled 10 parents of high-need children scheduled for dental treatment in the hospital operating room at the same dental school. A final sample of 10 parents of children visiting a public health clinic for reasons unrelated to dental health care was enrolled. To be eligible, all study subjects had to speak English and be parents or primary caregivers of children 3 to 5 years of age visiting the health care setting for the first time. The majority of respondents were mothers of the children. Most respondents were white (75%), followed by black (15%) and other races (10%). Similar to the health professionals, parents were asked to indicate the relevance of the 36 items to their child using the VAS. After parents responded to the self-completed questionnaire, a trained interviewer asked them whether they had difficulty in understanding the questions and if any of their dental experiences were not covered by these questions.

Using the domains identified by Jokovic and colleagues [6] as a guide we identified four descriptive domains for items included in the child impact section (symptoms, function, psychological, self-image/social interaction) and two domains for the family impact section (parent distress, family function). Total VAS scores, mean scores and standard deviations were calculated for each of the 36 items. The total score for each item was then subtracted from the corresponding mean, and divided by its standard deviation to obtain standardized scores. The items were then ranked in decreasing order of "importance" based on these standardized scores. To identify items for the final ECOHIS, the two highest ranked items in each of the six domains by at least two groups of respondents (i.e., parent respondents in the hospital, dental clinic or health department groups) were selected. In the *symptoms *domains, we selected only the highest-ranked question (about the child's pain experience) and excluded the three items relating to bleeding gums, mouth sores/ulcers and bad breath because parents did not consider them relevant to children of preschool age. Rankings for the seven items within the *function *domain differed among the three respondent groups. Thus, we retained the 4 highest-ranking items from each of the four groups (1. drinking hot or cold beverages, 2. difficulty eating some foods, 3. pronouncing any words, and 4. missing preschool, daycare or school). The items related to the child having trouble sleeping, and being irritable or frustrated in the *psychological *domain were selected from a total of four items. Finally, the two highest-ranking items from the child *self-image/social interaction *domain relating to whether the child "avoided smiling or laughing when around other children" and "avoided talking with other children" were retained.

On the family section of the ECOHIS, the items related to being upset and feeling guilty because of the child's dental problems or treatment experience ranked the highest in the parent *distress domain *from a total of 10 items in that domain. Similarly, in the *family function *domain, items related to taking time off from work and experiencing a financial impact on the family ranked the highest for at least two of the three groups of respondents from a total of five items.

### Testing

#### Pre-testing

The ECOHIS was administered to 6 parents of preschool age children in the department of pediatric dentistry at UNC to assess its readability, parents' ease of interpreting the lead-in and the scale itself, and the self-administered format of the scale. No changes needed to be made to the scale or its format of administration.

#### Validity and internal consistency reliability assessment

Validity and internal consistency reliability of the ECOHIS was assessed among a convenience sample (N = 295) of parents or caregivers of 5-year-old children from 5 high-income (median annual income = $40,872) and 3 low-income counties (median annual income = $26,459) in North Carolina who were selected for a larger population-based epidemiological study. Further details of the study sample have previously been published [22]. Parents responded to a self-completed 41-item questionnaire that included the 13-item ECOHIS and other questions relating to their child's oral health. Children also underwent an examination for dental caries and treatment experience by trained and standardized dental examiners.

#### Scoring and analysis of ECOHIS for validity and reliability assessments

Because of the infrequent nature of oral health problems and the young age of children being considered, the parent was asked to consider the child's entire life span when responding to the questions. Response categories for the ECOHIS were coded: 0 = never; 1 = hardly ever; 2 = occasionally; 3 = often; 4 = very often; 5 = don't know. ECOHIS scores were calculated as a simple sum of the response codes for the child and family sections separately, after recoding all "Don't know" responses to missing. For those with up to two missing responses on the child section or one missing on the family section, a score for the missing items was imputed as an average of the remaining items for that section. Using this criterion, it is possible for a respondent to be included in the analytic sample for one but not the other section of the ECOHIS. Parents with missing responses to more than two child items and one family item were excluded from the analysis. The score for the child and family sections have a possible range from 0 to 36 and from 0 to 16, respectively. In addition, we assessed the frequency of missing and "Don't know" responses to the 13 items.

#### Construct validity

Data on a smaller subset of parents with complete information for the child's dental examination (N = 186) were used to assess the validity (convergent and discriminant) of the ECOHIS.

*Convergent validity *was evaluated based on Spearman's rank order correlations: 1. between child and family ECOHIS scores and two subjective (dental and general) self-reported health measures; and 2. between the child and family sections of the ECOHIS. The global health rating question asked the parent, "In general, how would you rate the overall health of your child?" The dental health rating question asked, "In general, how would you rate the dental health of your child?" The response options for the two questions were: 1. = Excellent, 2. = Very Good, 3. = Good, 4. = Fair, and 5. = Poor. We hypothesized that a parent who reported higher scores on the two sections of the ECOHIS (indicating worse quality of life for child) would be more likely to rate the general and dental health of his or her child fair or poor. We also hypothesized that the child and family sections of the ECOHIS would be significantly correlated because parents' assessment of their child's oral health is likely to be closely related to parental perceptions of the effect of their child's oral health on their family.

*Discriminant validity *was evaluated by comparing ECOHIS scores for children with one or more decayed and/or treated teeth to those without any dental disease. We also examined the ability of ECOHIS to discriminate among children with varying levels of dental disease. Two hypotheses were tested using ANOVA: 1. Parents of children with dental disease and/or dental treatment experience would report higher ECOHIS scores (indicating worse OHRQL) than parents of children free of dental disease experience. 2. Among children with dental disease and/or dental treatment experience, those with more dental disease/treatment experience would have worse OHRQL. We expected these relationships to hold for both the sections of the ECOHIS.

#### Reliability

*Internal consistency reliability* was assessed on the full sample (N = 295) for each of the two sections using Cronbach's alpha. 

To assess *test-retest reliability*, the ECOHIS was administered on two occasions (separated by three weeks) to another convenience sample of parents (N = 55) of preschool aged children recruited from daycare centers. Test-retest reliability was assessed using the intraclass correlation coefficient (ICC) calculated by two-way analysis of variance [23] using data from respondents who reported no dental visits or change in their child's oral health status during the 3-week interval between initial and follow-up assessments (N = 46).

## Results

Table 2 displays the distribution of responses to the ECOHIS among the sample of parents from the population-based survey. The items related to pain, irritation, difficulty eating and smiling, and missing preschool or daycare was reported most frequently on the child impacts section. Items related to taking time off from work, feeling guilty and financial impact were reported frequently on the family impacts section of the ECOHIS. Parents reported more child impacts (58.03%) than family impacts (45.62%); about 42% and 54% parents reported no impacts (floor effects i.e., the lowest possible score of 0) on the child and family sections, respectively. No ceiling effects were observed for either of the two sections (i.e., scores of 36 and 16 on the child and family impact sections, respectively) (results not shown).

**Table 2:** ECOHIS responses in the survey of parents of 5-year-olds (N = 295)

**Impacts**	**Never or hardly ever** **N (%)**	**Occasionally, often, or very often** **N (%)**	**Don't know** **N (%)**
**Child impacts**			
How often has your child had pain in the teeth, mouth or jaws	245 (83.1)	48 (14.9)	6 (2.0)
How often has your child ....because of dental problems or dental treatments?			
had difficulty drinking hot or cold beverages	270 (91.5)	16 (5.4)	9 (3.1)
had difficulty eating some foods	263 (89.1)	23 (7.8)	9 (3.1)
had difficulty pronouncing any words	277 (93.9)	10 (3.4)	8 (2.7)
missed preschool, daycare or school	274 (92.9)	19 (6.4)	2 (0.7)
had trouble sleeping	278 (94.2)	14 (4.8)	3 (1.0)
been irritable or frustrated	265 (89.8)	27 (9.2)	3 (1.0)
avoided smiling or laughing	280 (95.0)	14 (4.7)	1 (0.3)
avoided talking	290 (98.3)	4 (1.4)	1 (0.3)
			
**Family impacts**			
How often have you or another family member......because of your child's dental problems or treatments?			
been upset	269 (91.3)	25 (8.4)	1 (0.3)
felt guilty	255 (86.5)	37 (12.5)	3 (1.0)
taken time off from work	234 (79.3)	59 (20.0)	2 (0.7)
How often has your child had dental problems or dental treatments that had a financial impact on your family?	257 (87.1)	35 (11.9)	3 (1.0)

Overall, less than 7 percent of the sample responded, "Don't know" to one or more items (results not shown). Parents responded with a "Don't know" most often for the questions related to difficulty drinking and eating and pronouncing any words on the child impact section. About 2.2 percent of parents reported "Don't know" to one question, and 1.6 percent to four or more questions. In contrast, slightly more parents (8.1%) had missing responses to one or more items. About 6.5 percent had missing on an average of more than three items. After excluding the "Don't know" responses and those with more than two missing items on child and one on the family section, the maximum number of impacts reported was 28 on the child impact section and 16 on the family impact section.

### Validity

Table 3 presents sociodemographic characteristics of the parents and disease status of children whose data was used to assess validity of the ECOHIS (N = 186). The child's mother was most often the proxy respondent, and low- and high-income families were equally represented in the sample. About three-quarters of the parents were white. About 37 percent of children had evidence of dental disease and/or treatment experience, and 16 percent had untreated dental decay. More parents rated their child's general health as 'excellent or very good' than their dental health (88.6% vs. 50.9%).

**Table 3:** Parent and child characteristics from the validity study (N = 186)

**Parent & child characteristics**	**Frequency**	**Percentage**
**Parent demographics**		
Relationship to the child		
Mother	161	87.5
Father	16	8.7
Guardian/Grandparent/Other	7	3.8
Race		
White	128	69.2
Black	51	27.6
Other	6	3.2
Education level		
High school or less	42	22.6
More than high school	144	77.4
Employment Status		
Employed full-time or part-time	143	76.9
Unemployed	43	23.1
Income level		
<$30,000 per year	82	49.1
≥ $30,000 per year	85	50.9
		
**Child's clinical disease status**		
Decayed, missing and filled teeth		
None	117	62.9
One or more	69	37.1
Decayed teeth		
None	159	85.5
One or more	27	14.5

Both hypotheses regarding convergent validity were confirmed. ECOHIS scores were statistically significantly correlated with the global dental and general health measures in the expected direction (Table 4). The correlation between the child and family impact sections was statistically significant (Spearman's r = 0.36, *P *≤ 0.001). Results of the assessment of discriminant validity indicated that overall, children with either 1–3 or ≥ 4 decayed and/or treated teeth had higher ECOHIS scores on both sections of the ECOHIS than those who were free of dental disease (Table 5). In addition, children with ≥ 4 decayed and/or treated teeth had significantly higher scores on the child, but not family section than those with 1–3 affected teeth.

**Table 4:** Findings for convergent validity of the ECOHIS

**Variable**	**Child section**	**Family section**
	*Spearman's r*	*Spearman's r*
General health rating	0.39**	0.20*
Dental health rating	0.41**	0.30**

* Statistically significant at *P *≤ .0001; ** Statistically significant at *P *≤ .001

**Table 5:** Findings for discriminant validity of the ECOHIS

	**Number of decayed and/or treated teeth**		
	**None**	**1–3**	**≥ 4**
**Child Impacts section**			
Sample size	112	37	32
Mean score	1.15	3.32	5.06
Std. Deviation	± 1.88	± 4.08	± 4.64
			
ANOVA comparisons tested	None vs. 1–3*		
	None vs. ≥ 4*		
	1–3 vs. ≥ 4*		

**Family Impacts section**			
Sample size	117	36	33
Mean score	1.0	2.75	3.82
Std. Deviation	± 1.65	± 3.05	± 3.96
			
ANOVA comparisons tested	None vs. 1–3*		
	None vs. ≥ 4*		
	1–3 vs. ≥ 4 NS		

* Statistically significant at *P *≤ .05, NS: Not significant

### Reliability

Cronbach's alphas for internal consistency reliability of items on the child and family sections were 0.91 and 0.95, respectively. The ICC for test-retest reliability was 0.84.

## Discussion

We have described the development of the Early Childhood Oral Health Impact Scale (ECOHIS) to assess the impact of oral health problems and related treatment experience on the quality of life of children 3 to 5 years of age and their families. The ECOHIS consists of child and family impact sections, with a total of 13 items. By using the input of health professionals and parents in the development process, we were able to identify items that were considered to be important by individuals closely involved in ensuring children's health and well being [18]. Oral disease and related treatment experience were found to measurably affect the oral health-related quality of life (OHRQL) of children and their families in this study. Further, respondents reported more impacts of these problems on the child compared to the parents or families.

We used global measures of oral and general health and actual disease status to examine the validity of the ECOHIS. These measures are commonly used subjective indicators that are highly correlated with clinically determined oral health status [24]. Statistically significant relationships between these indicators and ECOHIS scores provide evidence for convergent validity of the ECOHIS. As mentioned previously, many of the items in the ECOHIS are similar to those included in the Child Health Questionnaire (CHQ) and the Infant Toddler Quality of Life Questionnaire developed by Landgraf et al. (1996). Our findings of worse OHRQL among children with dental disease also are similar to those found for the relationship between general health issues and children's health related quality of life (HRQL) using the CHQ and the ITQOL, providing additional support for the relationship between disease and HRQL [20,21].

Evidence for discriminant validity of the ECOHIS is provided by the finding of higher ECOHIS scores (indicating worse OHRQL) among children who had clinically determined dental disease/treatment experience compared to those who were free from dental disease. The finding of higher ECOHIS scores on the child section among those with more than 4 teeth compared with children with fewer than 4 teeth with dental disease also provides evidence for discriminant validity. The lack of a significant difference in scores on the family section of the ECOHIS between children with high- and low- levels of dental disease is likely a function of the small sample size available for this assessment. However, the relationship between scores on the family section and the presence of dental disease is in the expected direction (higher ECOHIS scores and more dental disease). Further, the strong correlation observed between child and family items of the scale indicates that the ECOHIS has strong links with the underlying construct of OHRQL [19,25]. Additionally, the correlation between ECOHIS scores and the experience of dental disease substantiates existing evidence that parents can provide valid reports for their preschool children's OHRQL when these conditions are observable [8,26]. Cronbach's alphas of .91 and .95 for the child and family sections, respectively, indicate that the ECOHIS has excellent internal reliability [19]. The ICC of 0.84 indicates good agreement between the test and retest scores [27].

It is important to note that nearly half of the parents in this study reported no impact of oral health problems on their children's quality of life, leading to substantial floor effects. However, no ceiling effects were observed. The floor and ceiling effects for ECOHIS appear to be in accordance with the disease characteristics of the study sample wherein only a small percentage of the sample had dental disease. This distribution of scores also is characteristic of population-based studies where the disease distribution is likely to be skewed toward low levels of disease. However, the usefulness of the ECOHIS is evidenced by its ability to distinguish between those with and without dental disease experience, and to a lesser extent also among those with differing levels of dental disease or treatment.

About 7 percent of parents responded "Don't know" to one or more items on the ECOHIS. Jokovic et al. [4] stress the importance of including a "Don't know" response option in studies where respondents are asked to assess someone else's health or quality of life. We used a similar approach to the one described by Jokovic and colleagues (2003) of excluding these "Don't know" responses. In order to exclude them, the "Don't know" responses were recoded to missing, and then the criterion of excluding subjects with ≥ 2 and ≥ 1 missing responses on the child and family sections, respectively, was applied. However, in order to assess the potential bias that could be introduced by excluding these subjects, we assessed the percentage of the sample with such responses, and the items to which parents were more likely to respond with a "Don't know." As emphasized by Jokovic and colleagues [4], "Don't know" responses should be treated as a reflection of the construct being measured i.e., OHRQL, rather than a limitation of the scale.

The Michigan Oral Health-related Quality of Life Scale [8] was developed to measure the effects of early childhood caries (ECC) on the OHRQL of children using both parent and child self-reports. Because to our knowledge, it is the only other OHRQL scale currently available for use with preschool age children, we provide a comparison of the ECOHIS with that scale. The ECOHIS has several characteristics that distinguish it from the Michigan OHRQL Scale. Firstly, the Michigan OHRQL Scale was designed for use in a clinic setting and remains its only application. In contrast, the ECOHIS is intended for use in epidemiological surveys to assess the burden of dental disease and its treatment among young children at a population level. Because oral disease is a low frequency event at the population level, we asked parents or caregivers to consider the child's entire lifetime's experience of dental disease and treatment when responding to the ECOHIS. To capture this lifetime experience, we also used response options for the ECOHIS that assess the frequency with which oral disease and treatment affect a child's OHRQL. In contrast, the Michigan OHRQL Scale asks the parent how strongly he or she agrees or disagrees with statements about various aspects of their child's OHRQL, e.g., whether the child is currently experiencing pain or limitation of play because of his or her dental problems. Our approach allowed us to capture not only the occurrence of these experiences, but also the frequency with which such experiences affect children's OHRQL. Secondly, unlike the ECOHIS, the Michigan OHRQL Scale includes a child version to collect child self-reports of their OHRQL in a "Yes"/"No" format for questions similar to those on the parent version. However, Filstrup et al. [8] present little evidence that this approach provides valid and reliable assessments of the child's OHRQL. The authors do not report testing the child version of the scale for construct validity or reliability, and the descriptive analyses indicate only limited success of some questions from the scale in differentiating between older children with and without ECC. We believe that the measurement of child self-reports of their OHRQL is an area needing more research, and that currently there is little evidence to indicate that pre-school aged children can provide valid and reliable assessments of their own OHRQL. Lastly, unlike the Michigan OHRQL Scale, the ECOHIS assesses the effects of the child's oral health problems on not only the child, but also on his or her parent or caregiver. There is strong evidence in the literature that parents or caregivers of young children experience significant quality of life issues because of their children's health problems and treatment experiences [5,28,29]. The ECOHIS attempts to capture these effects related to children's oral health problems.

### Limitations, directions for future research and conclusion

Although we have provided evidence for the construct validity, and internal consistency and test retest reliability of the ECOHIS in assessing the effects of dental disease and treatment experience on young children and their families, these results should be viewed as preliminary. Data for the testing of the ECOHIS came from a convenience sample; therefore, our results provide evidence for its performance in this population only. Further, the validity of the ECOHIS was examined only among parents of 5-year-old children, and therefore needs to be tested among parents of children younger than five. The ECOHIS also needs to be further tested in different populations with known differences in clinical disease to further establish its discriminative properties in clinical populations. Dental disease and its treatment can have a negative influence on the quality of life of young children and their families. The assessment of these influences can help clinicians and researchers in their attempts to improve oral health outcomes for young children.

## Competing interests

The author(s) declare that they have no competing interests.

## Authors' contributions

BTP, GR and GS designed the study. BTP conducted the literature review and collected the data for the reliability study and with assistance from GR and GS analyzed and interpreted the data. BTP drafted the manuscript, with input from GR and GS. All authors read and approved the final manuscript.
